# Simulating gene trees under the multispecies coalescent and time-dependent migration

**DOI:** 10.1186/1471-2148-13-44

**Published:** 2013-02-18

**Authors:** Joseph Heled, David Bryant, Alexei J Drummond

**Affiliations:** 1 Department of Computer Science, University of Auckland, Auckland, New Zealand; 2 Allan Wilson Centre for Molecular Ecology and Evolution, New Zealand; 3 Department of Mathematics and Statistics, University of Otago, Dunedin, New Zealand

## Abstract

**Background:**

The multispecies coalescent model has become popular in recent years as a framework to infer a species phylogeny from multilocus genetic data collected from multiple individuals. The model assumes that speciation occurs at a specific point in time, after which the two sub-species evolve in total isolation. However in reality speciation may occur over an extended period of time, during which sister lineages remain in partial contact. Inference of multispecies phylogenies under those conditions is difficult. Indeed even designing simulators which correctly sample gene histories under these conditions is non-trivial.

**Results:**

In this paper we present a method and software which simulates gene trees under both the multispecies coalescent and migration. Our approach allows for both population sizes and migration rates to change over the species lifetime. Also, migration rates are specified in units of fraction of emigrants per time unit, which makes them easier to interpret. Overall this setup covers a wide range of migration scenarios. The software can be used to investigate properties of gene trees under different migration settings and to generate test cases for programs which infer species trees and/or migration from sequence data. Using simulated data we investigate the effect of migrations between sister lineages on the inference of multispecies phylogenies and on post analysis detection.

**Conclusions:**

Our results indicate that while estimation of species tree topology can be quite robust to the presence of gene flow, the inference and detection of migration is problematic, even with methods based on full likelihood models.

## Background

The multispecies coalescent model [1] is preferred to the ‘super-matrix’ method for phylogenetic inference when population sizes are large relative to the ages of the species being considered, because considerable differences are expected between individual gene trees and the species tree they evolve within [2,3]. This is understood both theoretically [2] and by simulation [3-5]. Recent developments have produced a number of methods and software packages for estimating species trees under the multi-species coalescent model [4-8]. Of these methods it is the full Bayesian implementations that are expected to perform the best as they use all available information and this is born out in simulation [5,9].

In all of these implementations, strict divergence is a standard assumption of the multispecies coalescent. Under strict divergence, a species is a perfectly mixing Wright-Fisher population until the moment of splitting, and from that point onwards the two sub-species evolve in total isolation. Strict divergence is a simplifying assumption, one which is violated by the presence of horizontal gene transfer, reassortment, migration or any other means of gene flow. Such simplifying assumptions are common in scientific models due to incomplete understanding of the processes involved, unavailability of analytical solutions or limitations in computational resources.

Here we focus on the effect of violating the central assumption of strict divergence. We model one specific type of gene flow – migration – and investigate its effects on the Bayesian inference of multispecies phylogenies. There are several software packages which infer species trees from multiple loci [4,7,10]. We explore the impact that migration has on this posterior distribution using the ⋆BEAST package [5].

Models of genetic differentiation in subdivided populations go back more than 70 years. In 1943 Wright introduced the “Island Model” in which “*the total population is assumed to be divided into subgroups, each breeding at random within itself, except for a certain proportion of migrants drawn at random from the whole*” [11]. Wright views the model as one extreme of the more general case of *Isolation by distance*, and also investigates an alternative, “local embedding in a continuous area”, where the population is distributed in a metric space and the probability of contact is inversely proportional to distance. Other intermediate models include Kimura’s “Stepping Stones” model [12,13] and the more general “Migration Matrix” which encapsulates geographic (or other) barriers to migration [14].

There are a large number of existing coalescent simulators [15-21] allowing for varying degrees of flexibility in modeling migration between related and unrelated populations. A common assumption behind the standard island models implemented in these existing simulators is that the rate of migration between two populations is either constant, or piecewise constant. If two populations only slowly become isolated after divergence then there will be a gradual decline in gene flow (migration), rather than a sudden drop. For this reason we extend the standard migration models to allow continuous change of migration rate over time. This modification adds some complexity to the simulation algorithms.

Given that the gradual decline of gene flow after divergence could well be a likely occurrence, we consider the effect this migration has on inference of species trees. It has been previously shown [22] that *IMα*[23,24] estimates are quite robust to moderate model violation, in which there is a “realistic” level of population structure within each species. However, IM *α* assumes that the species tree ranked topology is known, while this may be hard to pre-determine in many cases where migration is present. Here we examine the effect of migration on the posterior distribution of species trees without prior constraints on topology or divergence times.

Wright [25] showed that, in island models, it takes only one migrant per generation into each population (i.e. *Nm*>1) to prevent differentiation of two populations, given neutral markers [26]. Wright’s rule has clear implications for the inference of species trees, even with lower levels of migration. In order to test Wright’s rule for species trees, we wrote the simulator so that migration is determined in terms of the expected number of migrants, irrespective of population size. We then examine the relationship between the species tree as inferred from simulated sequence data and the true species tree.

## Implementation

### Model for two species with time-dependent migration rates and population sizes

**Figure 1:**
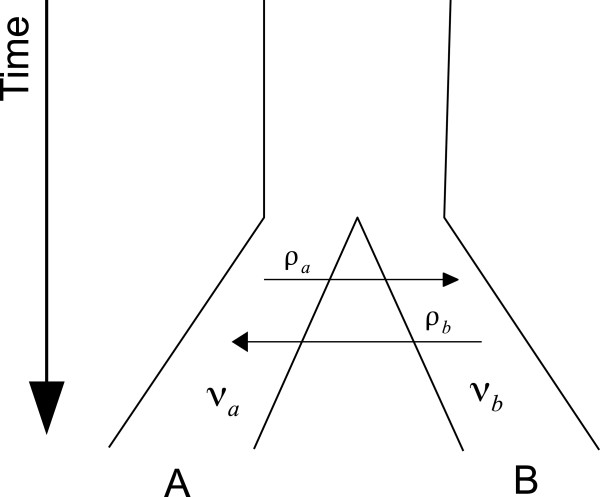
**Classical migration model for two populations.** Standard migration model for two populations. In the standard model, population sizes and migration rates are constant throughout the species time-span.

We begin by extending the two species model (Figure 1), allowing migration rates and effective population sizes to change over time. We then extend the model to any number of species under both the multispecies coalescent and the “Island Model”.

The model specifies how lineages from two species interact over time. Just like the coalescent, it is best viewed as going back in time. Starting at time zero (present) with *n*_*a*_ and *n*_*b*_ lineages from *A* and *B*, two lineages from *A* may coalesce at some past time, reducing *n*_*a*_ by one. Also, a lineage may “jump” from *A* to *B*, which corresponds to a migration event from *B* to *A* going forward in time. Obviously, coalescence of two *B* lineages and a “jump” from *B* to *A* are likewise possible.

The instantaneous rate at which coalescent events occur depends on the effective population size, *N*_*e*_(*t*). The rate of coalescence increases both when the effective population size decreases or when the generation time decreases. When specifying continuous models we typically leave generation time unspecified and use the function *ν*(*t*), the effective population size scaled by the generation length *τ* (i.e. *ν*=*N*_*e*_*τ*). For example, take *ν*(*t*)=0.1 over a species tree branch spanning time interval *t*=[0,1] when time is measured in millions of years; For a generation time of 1 year (*τ*=10^−6^ Myr) those translate to one hundred thousand individuals (i.e. *N*_*e*_=*ν*/*τ*=0.1/10^−6^) over one million years. If on the other hand time was measured in thousands of years, with a generation time of 1 year *τ*=10^−3^ the same parameter values would equate to 100 individuals over one thousand years. If time is measured in generations (*τ*=1), then *N*_*e*_ and *ν* are equal. Note that the properties of the model depend only on the ratio of population sizes and times; generation time is used only when converting time and population sizes into years and number of individuals.

The instantaneous rate of coalescence is 1/*ν*(*t*) and the density of two lineages coalescing at time *t* is [27,28],

(1) $$f(t)=\frac{1}{\nu (t)}\text{exp}\;\left(-\overset{t}{\underset{0}{\int }}\frac{\text{dx}}{\nu (x)}\right)$$

**Figure 2:**
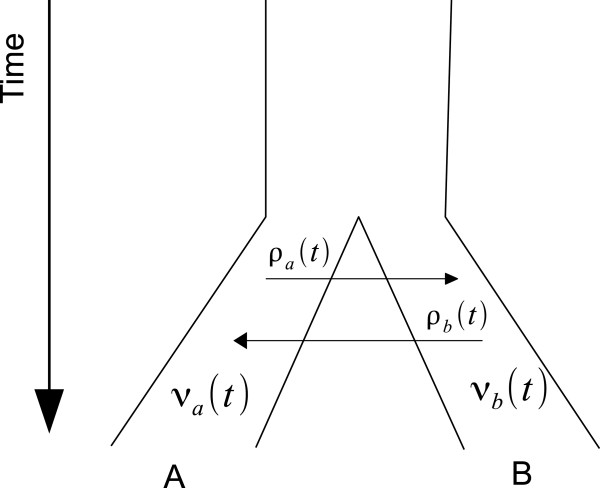
**Migration for time-dependent population sizes and migration rates for two populations.** Migration model for two populations where population size and migration rates vary over time. A migration rate of zero indicates complete separation.

Modelling two species with reciprocal migration requires two population functions, *ν*_*a*_(*t*) and *ν*_*b*_(*t*), and in addition two migration rate functions - *ρ*_*a*→*b*_(*t*) and *ρ*_*b*→*a*_(*t*) (Figure 2).

Migration rates are specified in terms of *m*_*a*→*b*_(*t*), the fraction emigrating out of A per unit time. The instantaneous rate *ρ*_*a*_(*t*) of migration from *A* to *B* at time *t* is *m*_*a*→*b*_(*t*)*ν*_*a*_(*t*). Under this parametrization the migration fraction has an easy to interpret intuitive meaning – the average number of individuals migrating in one generation per population unit. For example, an emigration fraction of *m*_*a*→*b*_(*t*)=1 means 100% of population A emigrates over one time unit.

It may seem that unequal migration rates would cause population sizes to change over time but this is not the case. The model is in fact an extension of the classic Wright-Fisher model; under Wright-Fisher the parent of every individual is chosen uniformly at random from all individuals in the previous generation. When migration is allowed, the ancestor of *a* from *A* may be one of the migrants *b* from *B*[29]. The instantaneous rate *f*_*a*→*b*_ for having an ancestor from the other population is the ratio of emigrants and effective population size,

(2) $$f_{a\to b}(t)=\frac{m_{b\to a}(t)\nu_{b}(t)}{\nu_{a}(t)}.$$

Since migration is a non-homogeneous Poisson process, the density of migration waiting time from *B* to *A* at time *t* can be derived from the rate (equation (17) in [27]),

(3) $$r_{a\to b}(t)=f_{a\to b}(t)\text{exp}\;\left(-\overset{t}{\underset{0}{\int }}f_{a\to b}(x)\text{dx}\right)$$

Equation (2) is the continuous equivalent of the “backward migration rate” (Lemma 1 in [30]). We follow Notohara, whose formulation has a fixed probability of migrating to another population for each individual in each generation. We think using the fraction of emigrants is better than using a fixed rate for modeling populations whose size may change over time.

### Migration under a species tree

**Figure 3:**
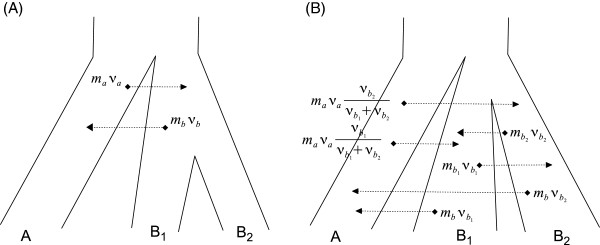
**Migration rates for a species tree.** (**A**) Migration between *A* and *B*. (**B**) Migration between A, *B*_1_ and *B*_2_.

The two-populations model can be extended to a species tree in a natural way. When population *B* splits into *B*_1_ and *B*_2_ there are six migration processes operating in parallel between the three populations; two between *B*_1_ and *B*_2_, two between *A* and *B*_1_ and two between *A* and *B*_2_ (Figure 3).

The total rate between *A* and *B*_1_∪*B*_2_ after the second split, going forward in time, is **as if** the two *B*’s were one population, and the emigrants from A are split based upon the relative sizes of *B*_1_ and *B*_2_. The same logic applies to additional splits.

**Figure 4:**
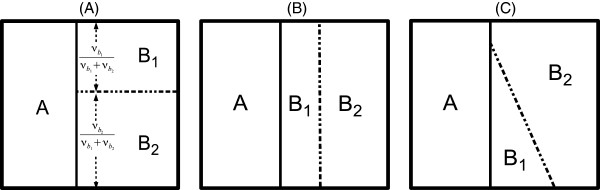
**Three possible effects of a split on migration.** (**A**) Graphical view of migration between *A* and *B*_1,2_ after the split of *B*. The ability of individuals from *B*_1_ or *B*_2_ to migrate to *A* (and vice versa) is exactly the same as before the split. (**B**) An alternative way for a split. Now, only migration between *B*_1_ and *A* is possible. (**C**) A second alternative showing uneven contact between *A* and *B*_1,2_.

Note that in principle there are many possible ways a split may affect the migration. Here, we assume that the split is B’s “internal affair” and that the ability of individuals to migrate is unaffected by the split (Figure 4A). But we can envision many other scenarios; for example, it may happen that the split has cut off one of the sub-species completely (Figure 4B), or any combination between those two options (Figure 4C). We do not investigate these other options in this manuscript, and only the first case is supported by the software.

### Drawing event waiting times for two populations

We begin by describing the simulation process for two sister lineages. With two species there are four possible events at any time, two coalescences and two migrations, each with its own rate. Since those processes are independent and memoryless, the waiting times starting at zero (now) and going back *T* time units can be drawn sequentially as follows, 

1. Start at time *t*=0 with *n*_*a*_,*n*_*b*_ lineages in populations *A* and *B*.

2. Independently draw waiting times for each possible event. Let *Δt* be the smallest waiting time.

3. Terminate if *t*+*Δt*>*T*.

4. Record the event with the smallest time. For example, if this is a coalescence in *A* then decrease *n*_*a*_ by one, and if this is a migration from *B* to *A* then going back in time we increase *n*_*b*_ by 1 and decrease *n*_*a*_ by 1.

5. Increase *t* by *Δt* and go to step 2.

Impossible events such as coalescence for less than two lineages or migration for zero lineages get infinite waiting time. When all the population and migration functions are constant this reduces to the classic model. In that case it is possible to draw a single number – the waiting time to the first event – instead of drawing all times as we do in step 2. This computational speedup is not available here since we let both population sizes and migration rates vary over time. Drawing the required waiting times is relatively straightforward using the classic inverse transform which can be applied to sample from any density *f*(*x*). Formulated as an equation for *t* we have:

(4) $$\overset{t}{\underset{0}{\int }}f(s)ds=-\text{lg}(1-U),$$

where *U* is a random number drawn from a uniform distribution on [0,1], and *f*(*s*) is any one of the rate densities. Numerical methods for computing the integral on the left and solving the equation are sufficient for the purpose of simulation, but with piecewise linear functions the integration can be done analytically. It is sufficient to show how to integrate and solve for linear functions, since the domain of any operation can be partitioned into sub intervals so that all the functions are linear on any sub interval.

On those sub-intervals, the migration fraction and both of the effective population size functions are linear, so the migration rate can be rewritten as follows,

(5) $$\frac{\left(a_{1}+b_{1}t)(a_{2}+b_{2}t\right)}{a_{3}+b_{3}t}=c_{0}+c_{1}t+\frac{c_{2}}{a_{3}+b_{3}t}$$

for suitable coefficients *c*_0_, *c*_1_ and *c*_2_. All those terms are easily integrated.

### Simulating a gene tree with migration under the multispecies coalescent

Simulating migration and coalescence for two species can be generalized to *n*_*s*_ species in a straightforward way. Again, we move back in time from the present (*t*=0) to the past. At any time point *t* there are *n*_*k*_ species with *l*_*i*_ lineages in species *i*. We generate nk+2nk2 waiting times, two migration times per pair of species, and one coalescence time per species provided *l*_*i*_>1. We pick the event with the smallest waiting time and apply it as previously explained, unless the event passes over a divergence time (i.e. a species union when going back in time). In that case the event is rejected, time is advanced to the divergence point, and the species lineages are merged, and the number of species is reduced by one.

### A simple parametrization of migration on a species tree

While a user can explicitly specify the migration rates for any species tree, doing so for more than a few cases is time consuming and prone to human bias. A specification via a more generic scenario where migration rates are set stochastically from a few parameters is more convenient and enables generating sets of test cases for quantitative exploration of the effect of migration on species inference.

One natural scenario is *gradual separation*, where migration has its highest rate at the time of divergence and declines to zero from that point forward in time. Gradual separation has two parameters, *M* and *S*. *S* is the average time between initial divergence and complete separation, as a fraction of the average species lifetime (typically 0≤*S*≤1). *M* is the migration fraction at the time of divergence *t*_*d*_, which declines linearly to zero at complete separation *t*_*s*_, that is, $m_{b\to a}(t)=m_{a\to b}(t)=\frac{M}{t_{d}-t_{s}}(t-t_{s})$ for *t*_*s*_≤*t*≤*t*_*d*_.

## Results

To quantitatively explore the effect of migration we generated several data sets using the gradual separation scenario. Unless stated otherwise, each set is composed of several test cases generated as follows: first draw a species tree at random using a Yule birth model with a rate *λ*=0.8; assign population sizes as explained in the Results section of [5], using an expansion factor 0.7 and standard deviation 0.4. Each divergence time is assigned its own migration interval; the interval length is drawn at random from a log-normal distribution with mean ^*S*^/_2*λ*_ and standard deviation 0.25 in log space, ^1^/_2*λ*_ being the expected length of the species tree branch in a Yule tree [31]. The time of complete separation for any clade is restricted to be earlier (when time flows forward) than any separation time of its descendants. That is, immigration between *A* and *B*_1_/ *B*_2_ is not allowed after immigration between *B*_1_ and *B*_2_ stops. Note that this is not a limitation in the model, and our software allows continued migration if required.

With the setup and methods as described, gene trees for 5 species with 10 individuals per species were simulated subject to coalescence and migration. The species tree has an average height of 1.6 $\left(\overset{5}{\underset{i=2}{\sum }}1/0.8i\right)$, which equals 8e-3 mutations when height is interpreted as millions of years and using a substitution rate of 0.005 per million years. The nucleotide average diversity on a random pair of taxa is 0.02, (diversity within species 0.0085, between species 0.022), the number of segregating sites for a 1600 bp sequence was 147(±27), and the number of haplotypes was 36(±3) out of 50.

### Migration events as a function of *M* and *S*

**Figure 5:**
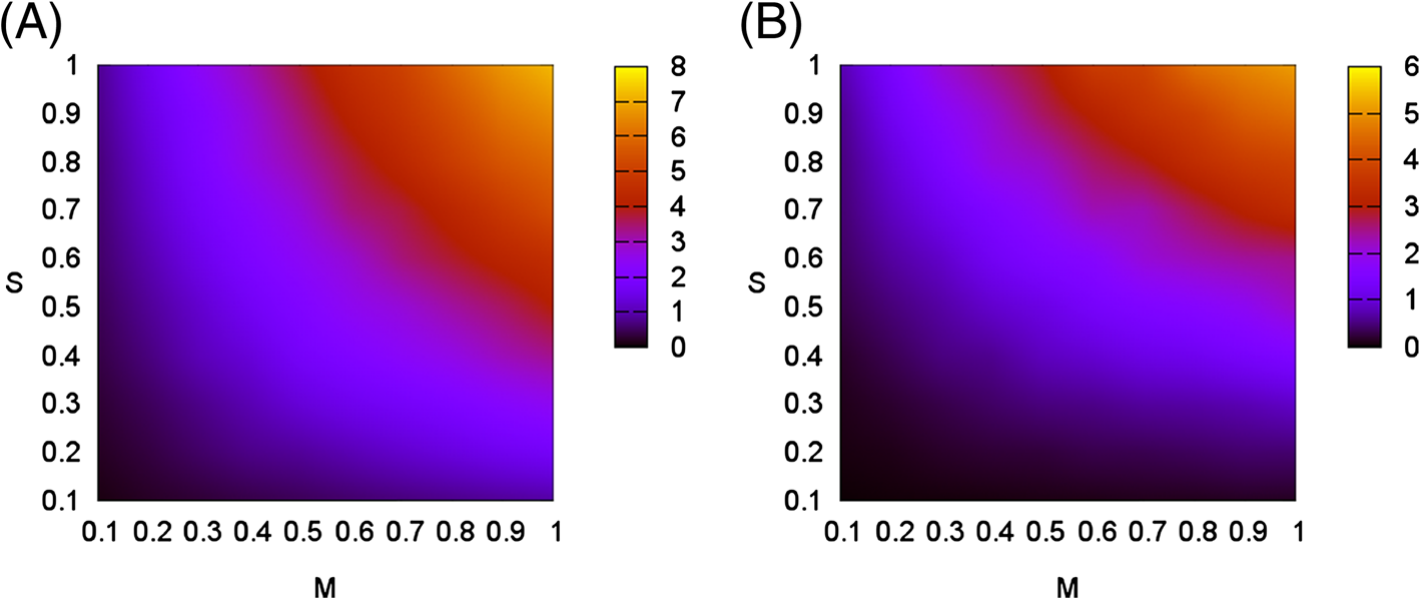
**Expected number of migration events.** (**A**) Expected number of migration events in one gene tree. (**B**) Expected number of gene tree coalescences inconsistent with the species tree.

How do values of *M* and *S* relate to actual migration events? Figure 5 shows the average number of migrations in the simulated genealogies as a function of *M* and *S*, under those particular settings. Figure 5 was generated by averaging the number of migrations in 100,000 simulations. Each simulation involved the generation of a species tree and a single gene tree “within” the species tree. The simulations were grouped into 1000 per point on the 10×10 grid in the unit square with a spacing of 0.1. Note that this is the actual number of migrations which occurred while generating the simulated gene tree, a number which is not generally available from real data.

The near symmetry around the *y*=*x* axis is not unexpected. Migration waiting times are exponential with a rate of $\int_{S}M$, which is equal to *M*×*S* for constant *M* and *S*. As a result, the number of migrations is dominated by the hazard value *M*×*S*, which is related to the probability of no migration occurring over an interval of duration *S* with a rate proportional to *M*.

In real multilocus sequence data, migration events are not observed directly – they alter the relation between the species tree and gene trees. Their effect can vary: a coalescence involving a migrating lineage can create an inconsistency between a gene tree and the strict species tree. Migrations not involved in such coalescence have a more subtle effect by altering coalescence waiting times. Note that the number of inconsistent coalescences (Figure 5B) is not symmetric with respect to *M* and *S*; the number of inconsistent coalescences increases faster with *S* than with *M*. In fact, the number of migrations or inconsistent coalescences have a good empirical fit to the form $\frac{MS^{c}}{\text{aM}S^{c}+b}$. Under our specific settings the number of migrations is roughly 8 to 10 times the hazard, and the number of coalescences is roughly 6 to 7.5 times the hazard.

Also note that those are expected values. With *M*=*S*=0.1, only 8% of the gene trees have migrations, while 27% have migrations for *M*=*S*=0.2. Since those gene trees are independent, the probability of not encountering a migration in a multilocus dataset drops exponentially with the number of loci.

### Weak and strong speciation

In the presence of migration there are several interpretations for the divergence times in inferred species trees. At one end, there is the *weak speciation tree*, where each divergence time marks the point at which species *begin* to separate. At the other extreme is the *strong speciation tree*, where each divergence time marks the point of complete separation. Software like *BEAST and BEST assume a model whereby the separation between species is instant and complete. What consequences does this model have on divergence time inferences when the speciation is only gradual?

To explore this issue, we simulated data for a range of M and S values. For each combination of parameters, we generated 100 replicate data sets, each set comprising 4 loci with 1600bp for 5 species, with 10 individuals per species. We generated samples from the posterior distribution using *BEAST. While BEST [7] and STEM [4] could, in principle, have also been used to generate samples from this distribution, *BEAST was more convenient for technical reasons. Our main interest is the impact of mispecification in the model used by all three packages, rather than a comparison of their sampling strategies.

For each data set we generated a chain of 8.8M trees, discarding 10% burnin, and then computed the posterior mean distance from trees in the sample to the weak and strong species trees respectively. We used the normalized rooted branch score of [5] when measuring distances between trees. A run is closer to the weak end if the distance to the weak tree is smaller than the distance to the strong tree. The table also shows the percentages for the same data set when using the simulated gene trees directly, effectively using “infinite” sequences instead of 1600bp.

Table 1 provides results for a few choice values of *M* and *S*. Each entry is the percentage of runs, out of a hundred, where posterior trees were closer to the weak end than the strong end. Table 2 shows two alternatives based on divergence time estimates. The first is the percentage of runs where the majority of divergence times were closer to the weak end. The second is the location where, on average, those divergence times are – that is, $\frac{t_{d}-t_{s}}{t_{w}-t_{s}}$ averaged over all posterior times, where *t*_*d*_,*t*_*s*_,*t*_*w*_ are the estimated divergence, divergence time in the strong tree and in the weak tree, respectively.

**Table 1:** Weak or Strong posterior trees?

**M**	**S**	**1600bp**	***∞*bp**
0.5	0.5	97%	85%
1	0.5	83%	71%
2	0.5	66%	43%
3	0.5	46%	30%
3	0.8	24%	12%

Percentage of test cases where posterior trees are closer to the weak speciation. Closer here means a smaller distance between trees based on the Rooted Branch Score. Shown are a few choice values of *M* and *S*, for both 1600bp sequences and “infinite” sequences (that is, using the simulated gene trees directly). Each entry is based on 100 independent test cases.

**Table 2:** Weak or Strong posterior trees: three measures

**M**	**S**	**Branch score**	**Pair divergence times**	**Mean pair location**
0.5	0.5	97%	90%	0.93
1	0.5	83%	91%	0.79
2	0.5	66%	67%	0.68
3	0.5	46%	57%	0.63
3	0.8	24%	37%	0.51

Three different measures assessing the relation of posterior samples from a ⋆BEAST run and their strong and weak species tree. Same data set as for Table 1. The first 3 columns are replicated from Table 1 for convenience. The forth column shows the percentage of test cases where the majority of divergence times were closer to the weak end. The fifth column shows the average location of divergence times when they are represented as a fraction between 0 and 1, 1 being the weak end and 0 the strong end.

One should keep in mind that there is not a single obvious way to match divergence times from the posterior to those from a fixed tree. The approach we took here is to use divergence times from all possible taxa pairs. This may lead to various types of bias which may depend on details of the species tree, or on the fact that there are more pairs with earlier divergence times than with later ones.

### Post analysis detection

The interplay between gradual speciation and divergence time estimation would be expected to have a significant impact on those methods using divergence times to test for gene flow and hybridization. One such method is JML, a program for detecting hybridization events using posterior predictive checking [32,33]. JML takes as input the posterior species trees from a ⋆BEAST analysis and the alignment from a single loci and outputs a list of pairs of species where it detects a possible migration.

To test the performance of JML in detecting gradual separation we generated 100 species trees for 5 species using a pure birth (Yule) process with a birth rate of 0.4. Population sizes were assigned randomly with a spread of ±20*%* around a mean of ^5^/_8_ (half of expected species lifetime ^1^/_2_×^1^/_2×0.4_). The same 100 species trees were used to generate two ⋆BEAST data sets, one without migration and the other with *M*=1 and *S*=^1^/_2_. Both sets used the same number of loci and individuals (4 and 6 respectively), The Jukes-Cantor substitution model with a strict clock with a rate of 0.005, and sequences of length 1600bp. With those settings, the sequences identity of two random individuals is on average 99.4%, while two individuals from different species are 97.1% identical, for the set of trees without migration [34].

⋆BEAST was run for each analysis and JML version 1.00 was run for each of the 4 loci with a significance level threshold of 0.1. JML detected migration in 63 cases out of the 100 for the first set (without migration), detecting 1 migration in 38 cases and 2 migration in 13 cases. JML detected migration in 69 cases out of the 100 in the second set. Out of a total of 887 pairs containing an inconsistent coalescence event occurring after the pair divergence, JML correctly detected 95 and falsely detected 48.

### Validation

The software code was tested extensively by comparing event time distributions from the code with distributions from a simpler process which proceeds backwards in time as follows: in each small time step *Δt* the rate of every possible event is multiplied by *Δt* to obtain the probability of that event occurring during the step. At most one event can occur in any single step.

**Figure 6:**
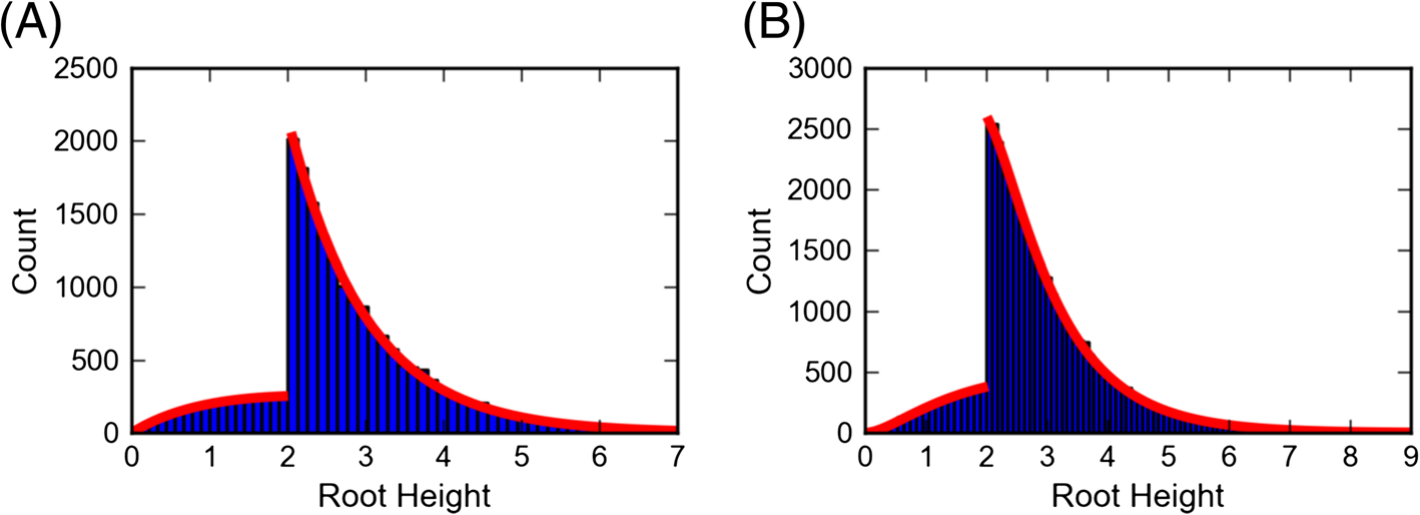
**Root Height distribution in two simple cases.** The distribution of the root height in two simple cases. The values from 20,000 simulated trees in shown in blue, while the theoretical values are shown by the red line. (**A**) 2 species *a* and *b* which diverged *T*=2 time units in the past, with a constant migration rate of 0.1. Population size is constant, *N*_*a*_=1, *N*_*b*_=2 and *N*_*ab*_=1. 1 lineage in each species. (**B**) Same settings, with 2 lineages from *a* and one from *b*.

Additionally we can derive the coalescence time distributions under basic settings, and compare those with the results from a large set of simulated trees. Figure 6 shows the distribution of the root height for two simple cases. Each case uses 2 species and constant population sizes and migration rates. To derive the theoretical distribution we use numerical exponentiation of the infinitesimal rate matrix *Q* (Equation 2.8 in [30]). exp(*QT*) provides the probabilities of the number of lineages and their location at time *T*. In the ancestral species we are reduced to the classical coalescent without migration, and the distribution for the root is simply the distribution of the sum of independent exponential processes with different rates. This distribution is given by equation 2.3 in [35].

## Discussion

It is somewhat surprising to find that ⋆BEAST detects incipient species before they are fully separated! Only at around 3 migrants per generation, over half of the species’ lifetime, the tide turns towards estimates of the species divergence times that reflect the respective times of complete species separation. This result does not seem to depend on the method used to measure the distance; the three measures shown in Table 2 show the same trend and are highly correlated. Given that ⋆BEAST assumes strict separation we were expecting posterior trees to tend to estimate complete separation times. This is not just an expectation: as the number of individuals and loci increase, posterior trees will inevitably get closer to estimating the times of complete separation. More loci and individuals mean more conflicting coalescences near the time of separation, and since divergence times are determined by the most recent common ancestor among all gene trees, those will get closer to the times of complete separation. However, as this table clearly indicates, for this to happen with a limited number of individuals and loci, high levels of migration may be required.

It is fairly obvious that small *M* and *S* values result in only few migrations, which affect only a few species divergence times. If most of the divergence times are unaffected, the tree as a whole will “support” divergence time estimates more reflective of incipient speciation times. But there is a second, deeper and less obvious reason, which may partially explain why posterior detection is ineffective. A model which co-estimates gene trees, divergence times and population sizes from fixed length sequence data can change any of the parameters to “explain” the model mismatch. The estimated divergence time for a species is bound from above by the time of the last ancestral lineage with descendants in all species. But those times are estimated from sequence data, and in the presence of a model mismatch any of the estimated parameters may be “pushed” to an incorrect value in a compromise to provide the best fit given the “wrong” model. It all depends on where the best overall likelihood is, given the model assumptions. It turns out that unless *M* and *S* are fairly large, more of the data supports weak speciation, and so coalescences due to migration are pushed back in time instead of species divergence times getting more recent. Actually, it is worse than that – even with large values of *M* and *S*, where most of the data supports a more recent divergence, the high sequence similarity allows a wide range in the genes divergence times, wide enough so that they can be spaced to match the times expected from that species divergence time with no migrations. That may explain the difficulty in detecting migration – the estimated gene trees exhibit very little model mismatch.

**Figure 7:**
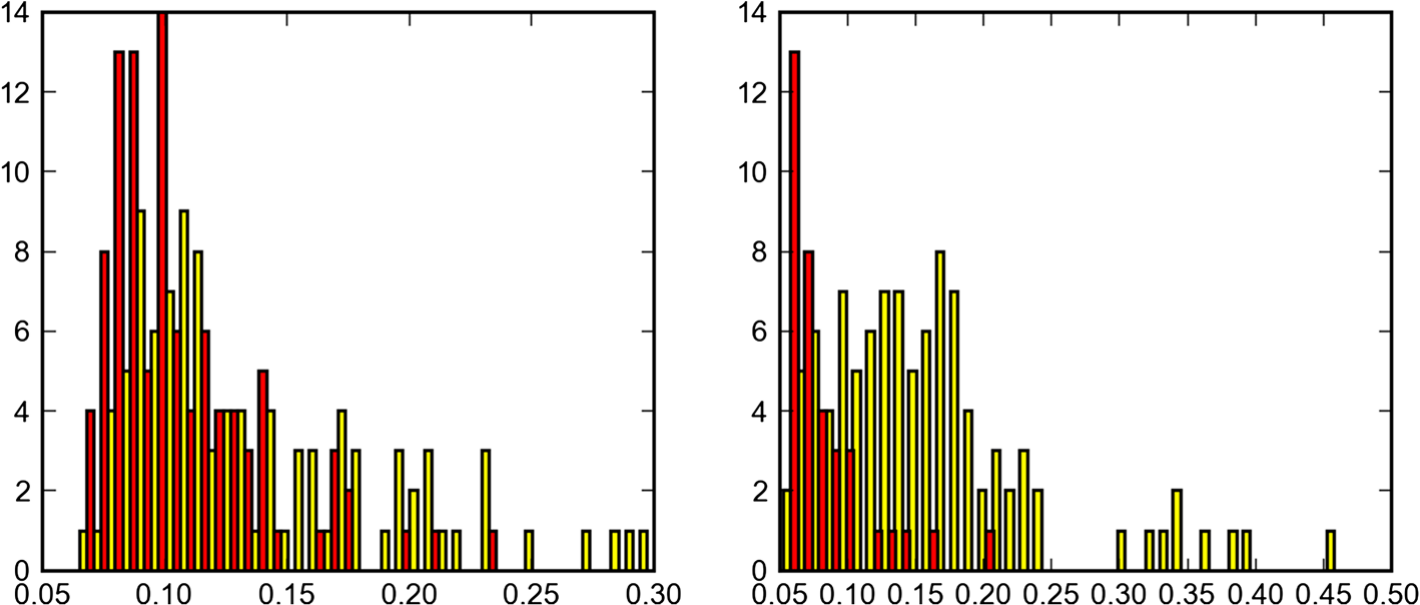
**Comparing distance distributions.** Comparing the distributions of posterior rooted branch scores of data sets with migration and without. The fundamental difference between short sequences (left) and infinite sequences (right) is clear.

Those observations may clarify the large difference between our analysis results for simulations of finite short sequences versus infinite length sequences. With infinite length sequences, coalescence events in gene trees are fixed in time, so we get estimates corresponding to complete separation times with smaller *M* and *S*. Figure 7 provides a visual reminder of how large those difference are. It shows the posterior estimate of the rooted score branch from the weak tree for 100 runs, with 1600bp and infinite length sequences. Note the almost complete overlap in the 1600bp distribution and the null distribution (no migration, in red), even though the two distributions are significantly different due to the longer tail of the (yellow) migration distribution.

When considering the results presented here we should keep in mind that migration can be modelled in many ways. We examined mainly gradual separation in species undergoing rapid radiation, where the amount of genetic diversity between sequences is relatively low. We have examined only an infinitesimal part of the problem domain. We used only a fixed birth rate, assigned population sizes in a particular way, considered only a few combinations of species, individuals and loci, and used *M* and *S* to model migration as linearly declining. It will be interesting to know how the results for this specific setup hold when we expand the domain in any direction, especially when considering a glaciation/warming-up type of model where species are totally isolated for a period of time and then allowed to reunite later, or when migration and population sizes are determined directly from an underlying geographic model. Nevertheless we anticipate the techniques presented here will prove useful in future research.

## Conclusions

We describe a technique for simulating genealogies according to the multi-species coalescent with time-dependent migration. Coalescent based simulators have the advantage of being more computational efficient than forward simulators, however the constraints of the coalescent sometimes make it more difficult to model complex evolutionary phenomena.

A key feature of our simulator is that it can incorporate variation in migration rates along the lifetime of a species. This is particularly important when exploring the dynamics of speciation, and the impact different forms of speciation have on the inference of species trees and demographics.

The complexity inherent in considering both (gradual) migration and incomplete lineage sorting necessitates an incomplete treatment of the problem. We have investigated only a tiny fraction of the parameter space that could be simulated. We have also not considered other inference packages (Bayesian or otherwise) that treat incomplete lineage sorting. The effects of gradual migration on these other methods remains to be determined. Some work has been done on the effect of migration on the estimation of species delimitation [36], but the authors did not address “gradual” migration and here too further research is required.

Our experimental results suggest, however, that inference of migration from observed data is difficult, even with a full-likelihood model. Even the simpler task of detecting migration is problematic, as demonstrated by JML “finding” migrations in approximately 2/3 of the test cases with and without migration. Naturally, the amount of signal will vary by context and the full extent to which parameters can be identified in practice remains unknown. Nevertheless, our initial observations signal the need both for caution and continued research. The simulation software we have presented here should provide a tool for this investigation.

## Availability and requirements

*Gene trees Under Migration Simulator* (GUMS) is part of biopy (http://code.google.com/p/biopy/), an open source, Python based bioinformatic package. See http://www.cs.auckland.ac.nz/∼yhel002/biopy/gums.html for instructions on using GUMS. Executable files for Linux and Mac OSX can be downloaded from the project download area (http://code.google.com/p/biopy/downloads/list). biopy is provided under the GNU GPL v3. license.

## Competing interests

The authors declare that they have no competing interests.

## Authors’ contributions

JH, DB and AJD designed the research plan. JH wrote the code, performed the analyses and wrote the first draft of the manuscript. All authors contributed to the final manuscript.
